# Solid Lipid Nanoparticles of Guggul Lipid as Drug Carrier for Transdermal Drug Delivery

**DOI:** 10.1155/2013/750690

**Published:** 2013-08-24

**Authors:** Praveen Kumar Gaur, Shikha Mishra, Suresh Purohit

**Affiliations:** ^1^Department of Pharmaceutics, I.T.S. Paramedical (Pharmacy) College, Muradnagar, Ghaziabad, Ultra Pradesh 201206, India; ^2^Department of Pharmacognosy & Phytochemistry, Jamia Hamdard, New Delhi 110062, India; ^3^Department of Pharmacology, Institute of Medical Sciences, Banaras Hindu University, Varanasi, Uttar Pradesh 221005, India

## Abstract

Diclofenac sodium loaded solid lipid nanoparticles (SLNs) were formulated using guggul lipid as major lipid component and analyzed for physical parameters, permeation profile, and anti-inflammatory activity. The SLNs were prepared using melt-emulsion sonication/low temperature-solidification method and characterized for physical parameters, in vitro drug release, and accelerated stability studies, and formulated into gel. Respective gels were compared with a commercial emulgel (CEG) and plain carbopol gel containing drug (CG) for ex vivo and in vivo drug permeation and anti-inflammatory activity. The SLNs were stable with optimum physical parameters. GMS nanoparticle 1 (GMN-1) and stearic acid nanoparticle 1 (SAN-1) gave the highest in vitro drug release. Guggul lipid nanoparticle gel 3 (GLNG-3) showed 104.68 times higher drug content than CEG in receptor fluid. The enhancement ratio of GLNG-3 was 39.43 with respect to CG. GLNG-3 showed almost 8.12 times higher *C*_max_ than CEG at 4 hours. The AUC value of GLNG-3 was 15.28 times higher than the AUC of CEG. GLNG-3 showed edema inhibition up to 69.47% in the first hour. Physicochemical properties of major lipid component govern the properties of SLN. SLN made up of guggul lipid showed good physical properties with acceptable stability. Furthermore, it showed a controlled drug release profile along with a promising permeation profile.

## 1. Introduction

**Figure 1:**
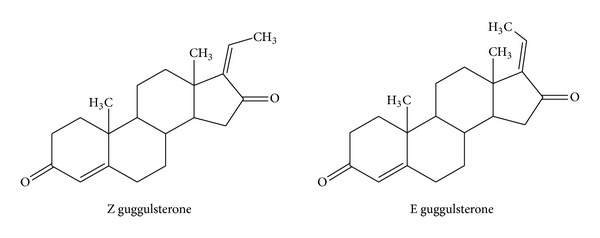
Structures of Guggul lipid components.

Guggul lipid is an ethyl acetate extract of guggul resin, obtained from *Commiphora wightii *(family: Burseraceae), and is official in Indian pharmacopoeia. The active constituent of Guggul lipid is guggulsterone (4,17(20)-pregnadiene-3,16-dione), which is present in a concentration of 4.0–6.0%. Guggul lipid contains a mixture of E and Z stereoisomers of guggulsterone. Among them, Z-isomer is potent antilipidemic. The structures of guggulsterone are quite similar to cholesterol (except the presence of the side chain) which is an important constituent of lipid-based formulation (Figure 1).

Addition of cholesterol in lipid-based formulations is known to enhance the stability [1–4]. Furthermore, there is a need to explore new lipid molecules to develop stable nanoparticles and effective drug delivery system. 

Lipid-based formulations constitute an important category and can be used to influence the absorption of active ingredients by means of modification of release of active ingredients. The biocompatibility of lipid-based carriers makes them attractive candidates for the formulation of pharmaceuticals. Solid lipid nanoparticles (SLNs) were developed in the early 1990s and have been considered to be promising drug carrier systems since then, especially with a view to give the incorporated active substance a sustained-release profile [5–8]. The main advantages of SLNs over other traditional drug carriers are good biocompatibility, lower cytotoxicity, drug targeting, drug release modulation, and the possibility of production on a large industrial scale [9].

Skin as an administration route offers advantages like ease of access avoidance of first pass metabolism and gastrointestinal disturbances; however, the selective permeability of skin presents the major hindrance with these attempts. The skin is composed of a dermis and an epidermis. Epidermis contains an uppermost layer of dead cells called stratum corneum (SC). In SC, corneocytes are surrounded by a cell envelope composed of cross-linked proteins and a covalently bound lipid envelope and are embedded in lipid lamellar regions, which are oriented parallel to the corneocytes surface. The SC lipids play an essential role in maintaining and structuring the lipid barrier which affords protection against external insults and water loss through the skin and is the reason of skin's selective permeability [10]. Several methods had been developed to enhance the transdermal drug permeation like the following physical methods: iontophoresis, electroporation, ultrasound, ablation, or chemical enhancers, for example, alcohols, terpenes, and azones [11–17]. 

In the present study, we developed an SLN formulation using Guggul lipid as main lipid component and diclofenac as a model drug and evaluated for physical parameters, transdermal drug permeation, stability, and anti-inflammatory activity. The developed formulations were compared with an established, commercial transdermal emulgel (CEG) containing diclofenac diethylammonium (Voltaren Emulgel). A carbopol gel formulation containing free diclofenac sodium (CG) was also prepared and evaluated for release. These results can prove to be useful in designing specific formulations for transdermal drug absorption.

Attama et al. formulated diclofenac sodium SLN for drug delivery to eyes by using a combination of homolipid from goat (goat fat) and phospholipid and observed that permeation of diclofenac sodium through the cornea construct was improved by SLN modified with phospholipid [18]. Shekar et al. formulated SLN of diclofenac for transdermal permeation using long-chain alkyl esters of *p*-amino benzoic acid (PABA) as possible new class of permeation enhancers [19]. Liu et al. formulated diclofenac sodium-loaded SLN by emulsion/solvent evaporation method [20]. Chime et al. prepared diclofenac potassium-loaded solid lipid microparticle using solidified reverse micellar solution and found them suitable for oral and parenteral administration [21].

## 2. Materials and Methods

### 2.1. Materials

Diclofenac Sodium was the gift sample from Asoj Soft Caps, Baroda, India, whereas Guggul lipid was purchased from Sami Labs Limited, Bangalore, Karnataka, India. Glyceryl monostearate (1-stearoyl-rac-glycerol), stearic acid (octadecanoic acid) and Poloxamer 188 (polyethylene-polypropylene glycol) along with all the other chemicals were of analytical grade and purchased from Sigma-Aldrich (New Delhi, India). Commercial formulation was Voltaren Emulgel (Novartis) containing 1.16% w/w diclofenac diethylammonium equivalent to 1% w/w diclofenac sodium.

### 2.2. Methods

#### 2.2.1. Nanoparticle Formulation

Melt-emulsion sonication and low-temperature solidification methods were used to prepare the nanoparticles as per the composition given in Table 1. Briefly, drug (1%) and lipid were dissolved in ethanol (10 mL) and heated up to the melting temperature of the lipid. Poloxamer 188 and double distilled water were mixed at 70°C and added to the melted oil phase. The resulting emulsion was initially stirred at 10,000 rpm for 10 minutes by mechanical agitation (Remi, New Delhi, India) and then sonicated using a probe sonicator for 15 minutes at 100 W amplitude to form a nanoemulsion which was rapidly immersed into icy water (0°) for solidification of nanoparticles. Then the dispersion was filtered through a membrane (Immobilon-P membrane, 0.45 *μ*m pore size, Millipore Pvt. Ltd., New Delhi, India) to exclude the particles larger than 0.45 *μ*m [19, 22].

#### 2.2.2. Size Distribution and Charge Characteristics

TEM in conjunction with negative staining using phosphotungstic acid was used to determine size and shape. A drop of the sample was placed over a copper grid 1% w/v solution of phosphotungstic acid was added and dried. Philips CM-10 (Acceleration voltage: 100 kV; magnification: 450,000x; cryoattachment) was used to analyze the samples. A total of 100 particles were measured and average values were reported.

Polydispersity indices and zeta potentials were determined employing photon correlation spectroscopy using Zetasizer NanoZS (Malvern Instruments, UK), equipped with a 4 mW He-Ne laser (633 nm). The formulations were suspended in phosphate buffer (pH 7.4) and then analyzed.

#### 2.2.3. Entrapment Efficiency

Entrapment efficiency was determined by subtracting the unentrapped drug fraction from total drug. SLN dispersion (0.2 mL) was dissolved in methanol (5 mL) followed by vortexing (CM-101 PLUS, Cyclomixer, Remi, New Delhi, India). The total amount of drug was estimated by HPLC assay after suitable dilution of resultant solution with methanol.

Ultrafiltration using centrisart (3500 rpm for 15 minutes) consisting of filter membrane (molecular weight cutoff of 20,000 Dalton) at the base of the sample recovery chamber was used for entrapment efficiency determination. The drug content was determined by HPLC of the aqueous phase [19, 22].

#### 2.2.4. HPLC Assay

Drug content was determined by using HPLC analysis. The instrument specifications were LC-10AT VP pump, an SIL-10AF autoinjector, an SPD-10A UV-VIS detector, and an SCL-10A VP system controller HPLC system (Shimadzu, Japan). The column specification was Shim-pack VP-ODS, 4.6 mm I.D. × 150 mm, 5 *μ*m *φ* (Shimadzu, Japan). The elution was done isocratically with methanol/water/acetic acid (80 : 20 : 0.5, v/v/v). The injection volume and flow rate were 20 *μ*L and 1.0 mL/min, respectively [20, 23]. 275 nm was taken as *λ*_max⁡_.

Calibration curve was drawn between concentration and peak area (2–40 ng/mL). The equation was *y* = 11256*x* + 544.9 (*R* ^2^ = 0.998), where *x* is the concentration and *y* is the peak area.

#### 2.2.5. In Vitro Drug Release through Synthetic Membrane

In vitro drug release was estimated using cellulose acetate synthetic membrane having a molecular weight cutoff of 12 KDa. Before the experiment, the membrane was equilibrated in buffer (pH 5.5) at 37 ± 0.5°C and placed in Franz diffusion cell (nominal surface area 3.14 cm^2^). Acceptor compartment was filled with buffer (pH 5.5), and 1 g formulation was applied onto the donor side. Aliquots were taken out at predecided time intervals, and drug content was estimated using assay. The volume was replaced with fresh buffer [24].

#### 2.2.6. Accelerated Stability Studies

The formulations showing optimum physical parameters were evaluated for their stability using accelerated stability conditions after storing the SLN at 40°C ± 2°C and 75 ± 5% relative humidity (RH) for 180 days [25].

#### 2.2.7. Gel Preparation

All the SLN formulations were formulated into gel using carbopol 934 (carboxyvinyl polymer). Suitable amount of carbopol 934 was dispersed in water to make 1% w/w dispersion and stirred using mechanical stirrer. Then, 0.5% v/v triethanolamine was added to neutralize the dispersion. The gel was kept overnight to allow the removal of any entrapped air. Finally, SLNs were added and drug concentration was kept at 1% w/w [26].

#### 2.2.8. Viscosity

Brookfield DV III ultra V6.0 RV cone and plate rheometer (Brookfield Engineering Laboratories, Inc., Middleboro, MA) was used to determine the viscosity of the gel formulations by means of spindle no. CPE40 at 25 ± 0.5°C [26].

#### 2.2.9. Ex Vivo Skin Permeation Studies

The experiment protocol was reviewed and approved by the Institutional Ethical committee. Full thickness human skin was obtained from plastic surgery patients. Skin was washed with Ringers' solution after removing subcutaneous fatty tissues with a scalpel. Then it was dried, packed in aluminum foil, and stored in a polyethylene bag at −20°C until further use.

For the experiment, skin was allowed to thaw (37°C) and cleaned with Ringers' solution. Then it was placed onto the Franz diffusion cell (nominal surface area 3.14 cm^2^). The diffusion cell was kept overnight for equilibration after filling acceptor compartment with buffer (pH 5.5). Then formulation was applied onto the skin surface (dosage: SLN formulation = 500 mg; CG and CEG = 1 g). Drug content was analyzed at predetermined intervals [24, 27].

#### 2.2.10. In Vivo Skin Permeation and Pharmacokinetic Parameters

 Twenty-four albino rats (8–10 weeks old and average weight 300 g), divided into four groups, were used for the study. The animals were kept under standard laboratory conditions (temperature: 25 ± 2°C; relative humidity: 55 ± 5%), in polypropylene cages with free access to standard laboratory diet (Lipton feed, Mumbai, India) and water *ad libitum*. For the experiment, the animals were anesthetized by i.v. injection of a combination of ketamine hydrochloride (75 mg/kg) and xylazine (5 mg/kg). Then abdominal area was washed with distilled water and hair on abdominal skin was trimmed off.

Group I received 1 g (1.16% drug) of CEG while the other three groups received 100 mg gel of (GMNG-3) GMS nanoparticle gel, (SANG-3) SA nanoparticle gel, and (GLNG-3) Guggul lipid nanoparticle gel, respectively. The formulations were applied in open containers glued to the skin by a silicon rubber (area 3.14 cm^2^). The blood samples (0.2 mL) were collected at predetermined time intervals till 24 hours and centrifuged at 5000 rpm for 20 min to separate the blood cells from plasma. Then plasma was stored at −21°C until drug analysis by using HPLC assay [28].

#### 2.2.11. Anti-Inflammatory Activity by Edema Inhibition

Anti-inflammatory activity of the SLN gels was determined by using carrageenan-induced rat paw edema method in Wistar albino rats against indomethacin (Positive control). The commercial formulation was used for demonstrative purpose only. The protocol was reviewed and approved by the Institutional Animal Ethical Committee. Thirty rats were divided into five groups of six rats. Group I received Indomethacin (10 mg/kg; p.o.), whereas group II received 1 g CEG. Groups III, IV, and V were administered GMNG-3/SANG-3/GLNG-3 = 100 mg formulation. Transdermal formulations were applied to the skin surface (3.14 cm^2^) in open containers glued to the abdominal skin by a silicon rubber. The untreated paw was considered as negative control. Animals were fasted for 24 h before the experiment with free access to water. Carrageenan suspension (1%) in saline was prepared 1 h before experiment, and 0.1 mL was injected into the plantar side of right hind paw of the rat. Treatments were applied 1 h before the carrageenan injection. The paw volumes were measured initially and at 1, 2, 3, 4, 5, and 6 h after carrageenan injection using digital plethysmograph. Percentage edema inhibition was calculated by using formula given in data analysis [29].

#### 2.2.12. Skin Irritation Studies in Human Subjects

Twenty-four males in four groups were used. All of them, properly educated about the procedure of the test, and consent forms were signed. The upper arm area was thoroughly examined for any irregularities. Also 5% w/v solution of sodium lauryl sulfate (SLS) was taken as positive control and untreated skin as negative control. Formulations were applied onto the skin and held with a bandage. After every 24 hours till seven days, the bandage was removed, skin was wiped with cotton, and observations, were made before fresh application of the treatment. Skin irritation was assessed by visual observations and scores were given as follows: 0, no reaction; 1, weak spotty or diffuse erythema; 2, weak but well perceptible erythema covering the total exposure area; 3, moderate erythema; 4, severe erythema with edema; 5, very severe erythema with epidermal defects (blisters, erosions, etc.) [30]. Treatments were applied as:

 Group I positive control (SLS treated)
 Group II GMNG-3
 Group III SANG-3
 Group IV GLNG-3.

#### 2.2.13. Data and Statistical Analysis

*Ex Vivo Skin Permeation Study*. The permeation parameters such as steady state drug flux (*J*_ss_), lag time (*T*_lag_), permeability coefficient through the membrane (*K*_*p*_), and diffusion constant within the membrane (*D*) were calculated from the ex vivo drug permeation data. The permeation profiles were constructed by plotting the cumulative amount of drug permeated versus time. The slope of the linear portion of the profile, determined by linear regression analysis, was *J*_ss_, whereas the *x*-intercept of the extrapolated linear region of the curve gives *T*_lag_. *D* was calculated from *T*_lag_ with known thickness of the permeation barrier (*h*), and *K*_*p*_ was determined by steady state drug flux and applied dose using following formulae [31–38].

(1) $$\begin{array}{c} K_{p}=\frac{J_{\text{ss}}}{C_{d}},\\ D=\frac{h^{2}}{6\cdot t_{\text{lag}}}, \end{array}$$

 where *D* = diffusion coefficient within the skin (cm^2^ h^−1^), *h* = diffusional path length, and *C*_*d*_ = initial drug concentration in donor compartment.

Enhancement ratio was calculated by using the following formula [39, 40]

(2) ER=(Permeability  coefficient  of  test  formulation∗Permeability  coefficient  of  CG)

(*test formulation = CEG, GMNG, SANG, and GLNG).

*In Vivo Drug Permeation and Pharmacokinetic Parameters*. Plasma concentration (*μ*g) versus time (h) profile was prepared, and peak plasma concentration (*C*_max⁡_) and time of its occurrence (*t*_max⁡_) were read directly from the respective profiles. Area under concentration time curve (AUC_0→*t*_) was calculated according to linear trapezoidal method using Graph pad Prism Version 4 [28].

*Anti-Inflammatory Activity*. Percentage of edema inhibition was determined using the following formula [29]:

(3) $$\begin{array}{c} \left(T_{c}-\frac{T_{t}}{T_{c}}\right)\times 100, \end{array}$$

where *T*_*c*_ = thickness of paw in control; *T*_*t*_ = thickness of paw in treatment group.

Data was expressed as mean of 3 values ± S.D. except for ex vivo studies and experiments involving live subjects where mean of 6 values ± S.D. was used for calculation. Statistical analyses were performed using the Graph pad Prism Version 4 software. Statistical comparisons were made using analysis of variance (ANOVA) or the paired *t*-test, where appropriate and statistical significance was set at *P* < 0.05.

## 3. Results

### 3.1. Physical Characterization

**Figure 2:**
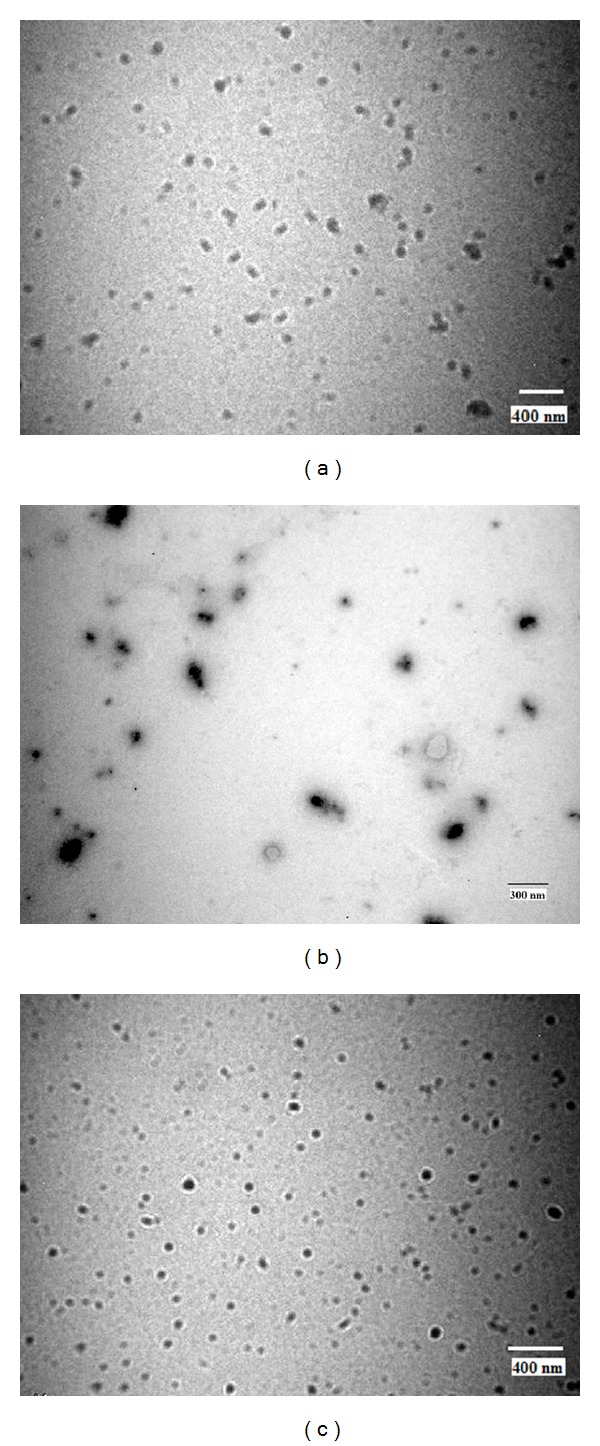
TEM photographs of (a) GMN-3, (b) SAN-3, and (c) GLN-3 formulations (×10000).

The nanoparticles were formed at all lipid compositions, and Figures 2(a), 2(b), and 2(c) show that nanoparticles were round and in size range of 98.12–137.6 nm. Minimum particle size was observed in nanoparticles prepared with Guggul lipid whereas, maximum particle size was found in SLN made with SA. The lowest polydispersity index value was 0.195 in GLN-3 containing the highest amount of Guggul lipid. The zeta potentials were in the range of −11 to −45 mV. Encapsulation efficiency showed an increasing trend with increasing amount of lipid in corresponding SLN formulations, maximum encapsulation being in GLN-3 (Table 2).

### 3.2. In Vitro Drug Release through Synthetic Membrane

**Figure 3:**
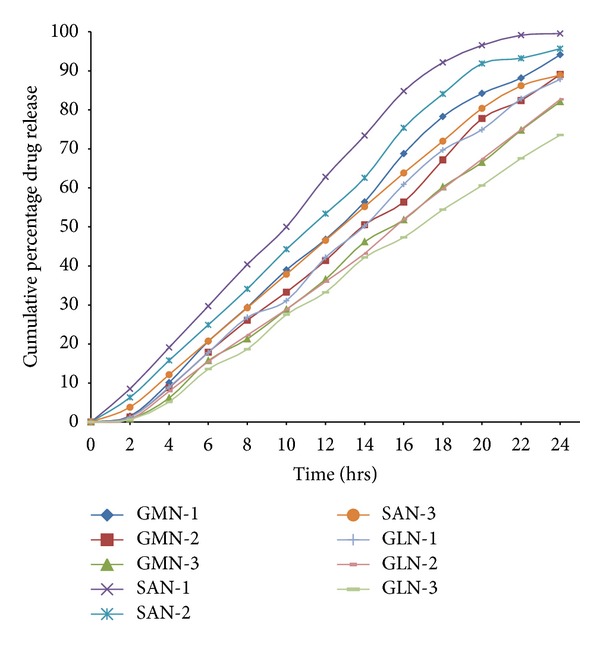
Release profiles of diclofenac from the SLN formulations in 24 hours through synthetic membrane.

Figure 3 shows the amount of drug released by formulations during the course of 24 h. The highest drug release was recorded in SAN-1 at 99.54%, while minimum drug release was found in GLN-3 at 73.54%. The values for drug release were in the range between 73.54–87.82%, 82.07–94.12%, and 88.89–99.54%, respectively, for GLN, GMN and, SAN formulations.

### 3.3. Stability Studies

The selected SLN formulations (GMN-3, SAN-3, and GLN-3) were evaluated for stability for 180 days. Most significant changes were observed for SAN-3 in particle size (74.5 nm), PDI (0.09), entrapment efficiency (12.93%), and drug release (7.54%), while GMN-3 showed moderate alteration in particle size, PDI, entrapment efficiency and in vitro drug release. The zeta potential of GLN-3 showed more reverse trend than that of GMN and SAN. The zeta potential of GMN-3 and SAN-3 became less negative while zeta potential for GLN-3 became more negative. The most stable formulation was found to be GLN-3 with almost negligible changes in physical parameters (Table 3).

### 3.4. Ex Vivo Skin Permeation Studies

**Figure 4:**
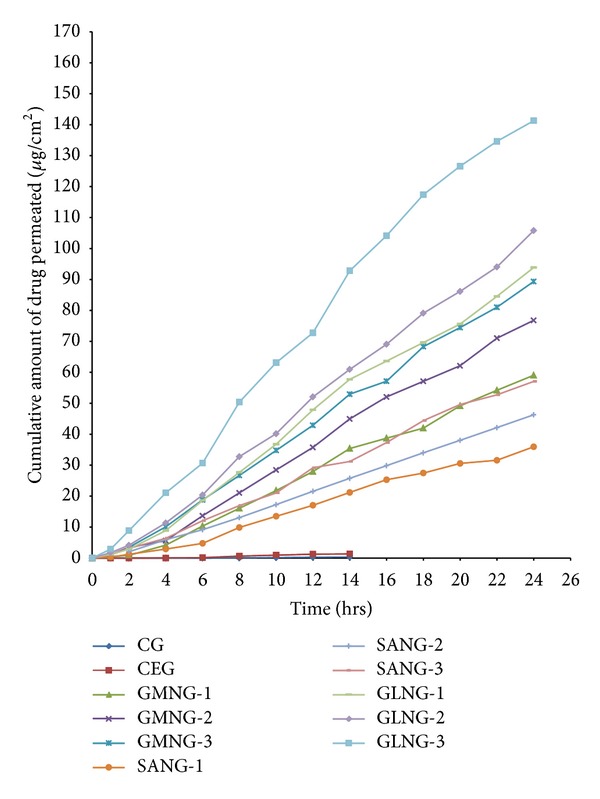
Ex vivo drug permeation in 24 hours; cumulative amount of drug permeated to receptor fluid through full thickness human skin.

Figure 4 shows the permeation profile of the drug from the gel formulations across the full thickness human skin in comparison with CG and CEG. SLN formulations showed the drug permeation up to 24 hours meaning that SLN prolonged the drug permeation, whereas CG and CEG showed drug permeation only upto 14 hours duration. Furthermore, the maximum permeation was observed in GLNG-3 (141.32 *μ*g/cm^2^). The drug permeation to the receptor fluid was the highest in GLNG formulations followed by GMNG and SANG formulations, respectively. Based on human skin permeation, steady state drug flux, lag time, permeability coefficient, diffusion parameter, and enhancement ratio were calculated and presented in Table 4. GLNG-3 showed the highest flux (6.363 *μ*g/cm^2^/h) and enhancement ratio (39.43) with respect to CG.

### 3.5. In Vivo Skin Permeation and Pharmacokinetic Parameters

**Figure 5:**
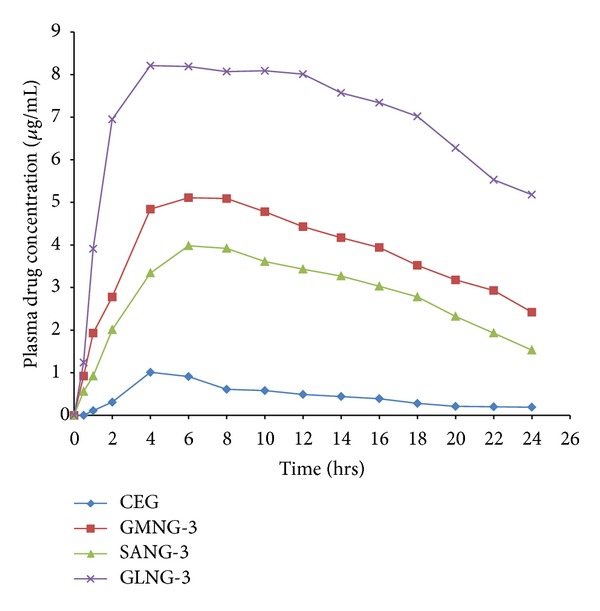
In vivo drug permeation profile of CEG and selected SLN formulations.

Based on drug release and skin permeation profiles, GMNG-3, SANG-3, and GLNG-3 were selected for pharmacokinetic comparison with CEG in albino rats (Figure 5). CEG showed maximum plasma concentration of 1.01 *μ*g at 4 h, while GMNG-3, SANG-3, and GLNG-3 gave *C*_max⁡_ of 5.11 *μ*g (6 h), 3.98 *μ*g (6 h), and 8.21 *μ*g (4 h). The AUC values of SANG-3, GMNG-3, and GLNG-3 were almost 6.26, 8.45, and 15.28 times higher than AUC value of CEG (Table 5).

### 3.6. Anti-Inflammatory Activity by Edema Inhibition

**Figure 6:**
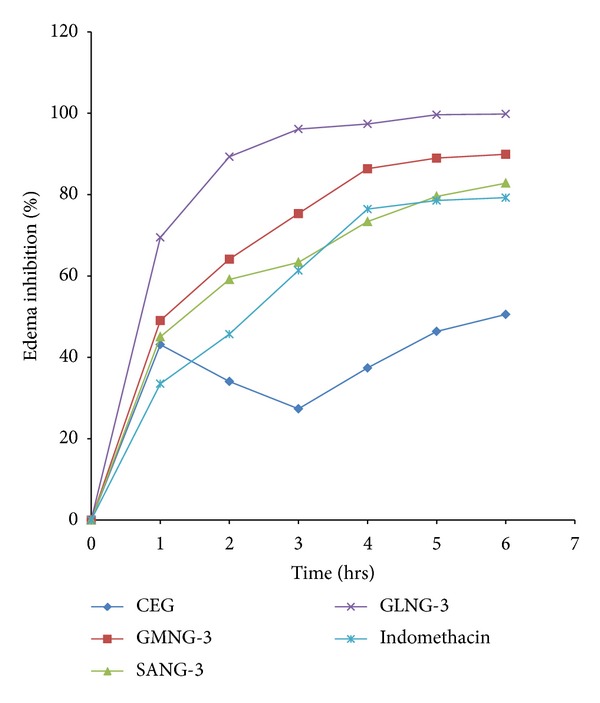
Percentage of edema inhibition by indomethacin, CEG, and selected SLN formulations.

The selected SLN formulations were evaluated for anti-inflammatory activity using carrageenan rat paw edema model against CEG and Indomethacin. GLNG-3 showed maximum edema inhibition at 99.83% in comparison with 50.54% and 79.25% edema inhibition of CEG and indomethacin (Figure 6).

### 3.7. Skin Irritation

All the groups treated with SLN gels have shown slight edema after 6-7 days. No group in SLN gel caused any erythema or any other dermal reactions (Table 6). All the SLN gel formulations have irritation index not more than 0.5.

## 4. Discussion

Solid lipid nanoparticles are an important carrier for drug delivery. In the present study, we have made SLN formulations of GMS, SA, and Guggul lipid for controlled delivery of drugs via transdermal application. 

GMS, SA, and Guggul lipid played the role of main lipid component in respective SLN formulation. GMS and SA contain a single hydrocarbon chain, whereas Guggul lipid is a planar molecule. Physical characterization of SLN, of abovementioned lipids reflects the effect of their structure. Particle size distribution shows that Guggul lipid SLN, were smaller than SLN made with either GMS or SA. The reason could be the stacking of lipid molecules to give a more compact nanoparticle. Polydispersity indices decreased with increasing content of lipid in each formulation category which means that increased lipid content yielded more uniformly sized SLN, regardless of the type of the lipid. Zeta potential is a product of surface charge and surface area. Smaller size SLNs usually yield more surface area than larger size SLN, for example, as the case with GLN formulations. However, SLN containing Guggul lipid was less negatively charged than SLN with either SA or GMS. That is because SA is 75% ionized at skin pH, and GMS yields SA residues which provide the negative charge. The encapsulation efficiency depends upon the amount of lipid phase. In each category, encapsulation efficiency increased with increased amount of lipid. Guggul lipid SLN showed highest encapsulation efficiency. This finding could be due to electrostatic repulsion of negatively charged lipid components in GMN and SAN formulations and negatively charged diclofenac molecule. During drug release study, SAN-1 showed the highest drug release followed by GMN-1. The formulation containing Guggul lipid showed controlled release of drug for 24 hours and even after 24 hours, GLN-3 retained almost 26.46% of drug. Usually smaller particles release higher drug content due to large surface area and low diffusional distance to be travelled by the drug molecule, but GLN formulations retain appreciable drug quantity despite being in lower size range which further enforces the possibility of better packing of drug in GLN formulations. GMN and SAN undergo lipid rearrangement causing drug expulsion which might be the reason of higher drug release in in vitro settings.

Based on the results from physical characterization and in vitro release, GMN-3, SAN-3, and GLN-3 were selected for stability evaluation which showed that GLN-3 was the least affected by the accelerated temperature and humidity conditions. The effect of accelerated condition was more pronounced on GMN-3 and SAN-3. Both of these lipids possess SA component. GMS is SA ester, while SA is by itself. Increased temperature promotes the clump formation which increases the particle size and PDI. The effect on particle size also reflected on zeta potential since effective surface area decreases with increase in particle size. SA, the main lipid of SAN-3, has the tendency of rearrangement in SLN which causes reduction in amount of entrapped drug. Storage at accelerated conditions makes the SLN unstable resulting in higher amount of drug release. Guggul lipid has shown more inertness than SA or GMS. Further, increase in drug release after storage at accelerated conditions might be due to recrystallization of lipid phase and expulsion of drug.

The formulations were then formulated into gels and evaluated in ex vivo drug permeation study using human skin which showed reversed trend in drug permeation. Even though the SAN and GMN released more drug content in drug release study, GLNG-3 corresponding to GLN-3 made highest drug content to permeate through skin into receptor fluid. Similar trend was observed in in vivo drug permeation studies as GLNG-3 gave considerably higher values for *C*_max⁡_ and AUC. It was an important observation that GLNG-3 showed the *T*_max⁡_ at 4 hours which was less than either GMNG-3 or SANG-3. Both GMN-3 and SAN-3 have shown greater drug release in in vitro drug release; however in skin permeation/in vivo studies, GLNG-3 has shown faster and higher drug permeation. In drug release study, the critical step is drug diffusion through the SLN matrix; however in skin permeation, rate limiting step is traversing the pathway through the skin. Guggul lipid has a planar structure and a log*P* value of 4.4 which helps in permeating through highly hydrophobic SC. In GLNG-3, plasma drug concentration remained in plateau range between 4 and 12 h making it a suitable controlled release formulation. In anti-inflammatory activity determination, GLNG-3 showed highest edema inhibition by virtue of higher quantity of permeated drug. None of the SLN formulations showed any potential irritant reaction except slight edema. According to Draize et al., formulations having scores of 2 or less are considered nonirritant [41].

Among all the lipids tested, Guggul lipid possesses anti-inflammatory activity of its own [42]. SLN made up of Guggul lipid showed good physicochemical parameters along with good stability and permeation.

## 5. Conclusion

SLN is an important mode of drug delivery, and in the present study three different lipids were evaluated for SLN formulation. Based on the results, it can be concluded that these SLNs showed optimum physical characteristics and permeation profile, promising stability, and good compatibility with skin. The most promising formulation was found to be GLNG-3 containing the highest quantity of Guggul lipid among all formulations. We suggest that Guggul lipid nanoparticles would be advantageous for controlled transdermal delivery of drugs.

## Figures and Tables

**Table 1:** Lipid composition for SLN formulations (Drug content 1%).

Lipid	Formulation code	Concentration (%)
GMS	GMN-1	2.5
	GMN-2	5
	GMN-3	7.5

SA	SAN-1	2.5
	SAN-2	5
	SAN-3	7.5

Guggul lipid	GLN-1	2.5
	GLN-2	5
	GLN-3	7.5

GMN: glyceryl monostearate (GMS) nanoparticles, SAN: stearic acid nanoparticles, GLN: Guggul lipid nanoparticles.

**Table 2:** Physical characterization of SLN and corresponding gels.

Lipid	Formulation Code	Size^∗#^ (nm)	PDI*	*ζ* potential* (mV)	Entrapment efficiency* (%)	Viscosity^†^ (Cps)
GMS	GMN-1	101.2 ± 3.2	0.35 ± 0.043	−29 ± 2.36	47.24 ± 3.94	18560 ± 23.4
	GMN-2	111.5 ± 3.9	0.34 ± 0.056	−27 ± 3.15	56.62 ± 2.41	18753 ± 21.2
	GMN-3	124.2 ± 4.1	0.31 ± 0.028	−23 ± 3.54	69.96 ± 3.61	18350 ± 20.7

SA	SAN-1	116.3 ± 4.3	0.48 ± 0.064	−45 ± 4.32	39.12 ± 4.72	16234 ± 19.3
	SAN-2	128.9 ± 5.1	0.45 ± 0.055	−40 ± 3.97	48.37 ± 3.21	16587 ± 24.8
	SAN-3	137.6 ± 6.23	0.40 ± 0.048	−36 ± 4.15	59.32 ± 3.59	16628 ± 21.5

Guggul Lipid	GLN-1	98.12 ± 1.2	0.22 ± 0.028	−11 ± 1.32	65.12 ± 1.32	18123 ± 12.1
	GLN-2	109.5 ± 1.5	0.21 ± 0.039	−13 ± 1.23	76.43 ± 1.71	18312 ± 14.5
	GLN-3	117 ± 1.7	0.2 ± 0.011	−15 ± 1.43	89.54 ± 1.43	19401 ± 11.8

*Determinations performed on SLN.

^†^Viscosity determined on corresponding carbopol gels.

^#^Size after extrusion.

All data expressed as mean ± S.D.; *n* = 3. *P* ≤ 0.05.

**Table 3:** Physical characterization of SLN after stability studies at 40°C ± 2°C and 75% ± 5% RH.

Physical characterization Parameters	Days	Formulation Code		
		GMN-3	SAN-3	GLN-3
Size (nm)	0th	124.2 ± 4.1	137.6 ± 6.23	117 ± 1.7
	30th	126.3 ± 4.6	189.4 ± 5.19	117.8 ± 1.4
	90th	136.8 ± 3.2	198.2 ± 5.64	119 ± 2.6
	180th	154.2 ± 5.4	212.1 ± 4.14	126 ± 3.2

PDI	0th	0.31 ± 0.028	0.40 ± 0.048	0.22 ± 0.028
	30th	0.32 ± 0.021	0.41 ± 0.032	0.22 ± 0.071
	90th	0.38 ± 0.035	0.42 ± 0.059	0.23 ± 0.087
	180th	0.39 ± 0.024	0.49 ± 0.035	0.26 ± 0.045

*ζ* potential (mV)	0th	−23 ± 3.54	−36 ± 4.15	−15 ± 1.43
	30th	−22 ± 3.76	−34 ± 4.52	−16 ± 1.21
	90th	−23 ± 3.18	−30 ± 4.98	−16 ± 1.76
	180th	−20 ± 4.25	−28 ± 3.17	−18 ± 1.65

Entrapment efficiency (%)	0th	69.96 ± 3.61	59.32 ± 3.59	89.54 ± 1.43
	30th	68.21 ± 4.41	58.87 ± 3.59	89.19 ± 3.32
	90th	67.45 ± 3.13	54.64 ± 3.59	88.34 ± 2.43
	180th	61.18 ± 3.23	46.39 ± 3.59	86.42 ± 1.46

In-vitro % cumulative drug release (in 24 h)	0th	82.07 ± 1.78	88.89 ± 2.34	73.54 ± 1.76
	30th	83.21 ± 1.57	90.48 ± 2.25	74.23 ± 2.23
	90th	85.43 ± 2.32	94.23 ± 2.92	75.12 ± 1.87
	180th	92.45 ± 3.97	96.43 ± 3.54	76.32 ± 2.13

All data expressed as mean ± S.D.; *n* = 3; *P* ≤ 0.05.

**Table 4:** Permeation parameters of the CEG, CG and Different SLN formulations.

Formulation code	Flux (*μ*g/cm^2^/h)	Lag time (h)	Permeation coefficient (cm/h × 10^−3^)	Distribution coefficient (cm^2^/h × 10^−3^)	Enhancement ratio
CG	0.317	2.8	0.0317	1.25	1
CEG	1.074	2.3	0.0925	1.56	2.917
GMNG-1	2.661	1.675	0.532	2.089	16.78
GMNG-2	3.43	1.475	0.686	2.372	21.64
GMNG-3	3.94	1.17	0.788	2.991	24.85
SANG-1	1.63	1.9	0.326	1.842	10.28
SANG-2	2.03	1.5	0.406	2.333	12.8
SANG-3	2.529	1.3	0.505	2.692	15.93
GLNG-1	4.128	1.1	0.825	3.1	26.02
GLNG-2	4.591	1.05	0.918	3.3	28.95
GLNG-3	6.363	0.4	1.25	8.7	39.43

CG: Carbopol gel (Conataing 1% Diclofenac Sodium).

CEG: Comercial Emulgel.

**Table 5:** Pharmacokinetic parameters of the CEG and selected SLN formulations.

Formulation code	*C*_max⁡_ (*μ*g/mL)	*T*_max⁡_ (h)	AUC (*μ*g · hr/mL)
Commercial gel*	1.01 ± 0.087	4	10.98 ± 0.039
GMNG-3	5.11 ± 1.07	6	92.8 ± 1.012
SANG-3	3.98 ± 1.42	6	68.74 ± 1.49
GLNG-3	8.21 ± 1.34	4	167.8 ± 1.24

*1 g gel formulation equivalent to 11.6 mg Diclofenac Diethylammonium for commercial gel (10 mg Diclofenac sodium).

^†^100 mg gel formulation equivalent to 1 mg of Diclofenac sodium for GLNG-3,GMNG-3 and SANG-3.

All data expressed as mean ± S.D.; *n* = 6; (*P* ≤ 0.05).

**Table 6:** Irritation score in human subjects.

Sub. no.	Positive control		GMNG-3		SANG-3		GLNG-3	
	Erythema	Edema	Erythema	Edema	Erythema	Edema	Erythema	Edema
1	4	4	0	1	0	0	0	0
2	3	4	0	0	0	1	0	0
3	4	3	0	1	0	1	0	1
4	4	2	0	1	0	0	0	0
5	3	4	0	0	0	0	0	1
6	4	3	0	0	0	0	0	1

Total			0	3	0	2	0	3

Average	3.67 ± 0.51	3.33 ± 0.81	0	0.5 ± 0.54	0	0.33 ± 0.51	0	0.5 ± 0.54

## Abbreviations

CEG: Commercial emulgel
CG: Plain carbopol gel containing drug
GLN: Guggul lipid nanoparticle
GLNG: Guggul lipid nanoparticle gel
GMN: GMS nanoparticle
GMNG: GMS nanoparticle gel
GMS: Glyceryl monostearate
HPLC: High performance liquid chromatography
KDa: Kilo Dalton
PDI: Polydispersity indices
RH: Relative humidity
SA: Stearic acid
SAN: Stearic acid nanoparticle
SANG: Stearic acid nanoparticle gel
SC: Stratum corneum
SLN: Solid lipid nanoparticle
TEM: Transmission electron microscopy.
